# Delta-like 4 is required for pulmonary vascular arborization and alveolarization in the developing lung

**DOI:** 10.1172/jci.insight.134170

**Published:** 2021-04-08

**Authors:** Sheng Xia, Heather L. Menden, Nick Townley, Sherry M. Mabry, Jeffrey Johnston, Michael F. Nyp, Daniel P. Heruth, Thomas Korfhagen, Venkatesh Sampath

**Affiliations:** 1 Division of Neonatology, Department of Pediatrics, Children’s Mercy Hospital, Kansas City, Missouri, USA.; 2 Genomic Medicine Center, Children’s Mercy Hospital, Kansas City, Missouri, USA.; 3 Division of Neonatology, Department of Pediatrics, Cincinnati Children’s Hospital, Cincinnati, Ohio, USA.

**Keywords:** Pulmonology, Vascular Biology, Endothelial cells, Molecular biology

## Abstract

The molecular mechanisms by which endothelial cells (ECs) regulate pulmonary vascularization and contribute to alveolar epithelial cell development during lung morphogenesis remain unknown. We tested the hypothesis that delta-like 4 (DLL4), an EC Notch ligand, is critical for alveolarization by combining lung mapping and functional studies in human tissue and DLL4-haploinsufficient mice (*Dll4^+/lacz^*). DLL4 expressed in a PECAM-restricted manner in capillaries, arteries, and the alveolar septum from the canalicular to alveolar stage in mice and humans. *Dll4* haploinsufficiency resulted in exuberant, nondirectional vascular patterning at E17.5 and P6, followed by smaller capillaries and fewer intermediate blood vessels at P14. Vascular defects coincided with polarization of lung EC expression toward JAG1-NICD-HES1 signature and decreased tip cell-like (*Car4*) markers. *Dll4^+/lacZ^* mice had impaired terminal bronchiole development at the canalicular stage and impaired alveolarization upon lung maturity. We discovered that alveolar type I cell (*Aqp5*) markers progressively decreased in *Dll4^+/lacZ^* mice after birth. Moreover, in human lung EC, *DLL4* deficiency programmed a hypersprouting angiogenic phenotype cell autonomously. In conclusion, DLL4 is expressed from the canalicular to alveolar stage in mice and humans, and *Dll4* haploinsufficiency programs dysmorphic microvascularization, impairing alveolarization. Our study reveals an obligate role for DLL4-regulated angiogenesis in distal lung morphogenesis.

## Introduction

Mammalian lung development progresses sequentially through the embryonic, pseudoglandular, canalicular, saccular, and alveolar phases (1). In preterm infants, exposure to noxious stimuli, such as infection, mechanical ventilation, and hyperoxia, disrupts lung development, resulting in bronchopulmonary dysplasia (BPD) (2, 3). Vascular maldevelopment is characterized by defects in pulmonary vascular growth and dysmorphic arborization, both of which are hallmarks of BPD (4, 5). The molecular mechanisms regulating distal lung vascularization, a requisite for alveolarization, are not fully understood, limiting our understanding of defective vascularization in BPD. Vasculogenesis and angiogenesis are distinct mechanisms that govern vascular network development. Based on vascular casts and electron microscopy of mouse lungs demonstrating initial discontinuity, followed by fusion of proximal and distal lung vasculature, deMello et al. suggested that vasculogenesis is important for peripheral pulmonary blood vessel development (6, 7). Parera et al. mapped the endothelial cell–specific *Tie2* promoter during lung development and suggested that angiogenesis is required for distal lung vascular arborization (8). However, the functional contribution of angiogenesis or vasculogenesis to lung vascular development and alveolarization (1, 9) has not been investigated. Moreover, the key endothelial cell (EC) molecular players that regulate angiogenesis and pulmonary vascular arborization required for normal alveolar development remain unknown.

During sprouting angiogenesis, expression of delta-like 4 (DLL4) in EC, induced by a VEGF gradient specifies a tip cell EC phenotype. Tip cells specify a stalk cell EC phenotype in adjoining EC, through transactivation of NOTCH-dependent signaling that inhibits DLL4 expression (10). Both homozygous and heterozygous deficiency of *Dll4* result in embryonic lethality between E9.5 and E11.5 in C57BL/6 mice with the absence of large vitelline vessels in yolk sac and reduced dorsal aorta (11, 12). During retinal vascular development, EC-specific *Dll4* deficiency leads to upregulation of tip cell–enriched genes, followed by more tip cells and vessel branches (13), whereas more DLL4 expression represses sprouting angiogenesis, EC proliferation, and migration (14). Besides angiogenesis, Dll4/Notch signaling maintains arterial identity of blood vessels, with *Dll4* deficiency reducing arterial vascular lumen diameter in embryos (11, 15). Although these studies evince a critical role for DLL4 in embryonal and retinal vascular development, the contribution of DLL4 to postnatal organ development, specifically lung vascularization and alveolarization, remains unknown.

Although the contribution of VEGF to lung development is established, the EC-specific molecular mechanisms that regulate distal lung vascularization and alveolarization have yet to be characterized (16–18). Based on our initial studies showing DLL4 expression in the mouse and human lung we hypothesized that DLL4 is required for pulmonary microvascular growth and arborization during distal lung morphogenesis, and *Dll4* deficiency will impair alveolarization. In this study, we used *Dll4^+/lacZ^* mice (CD1 strain) lacking 1 copy of *Dll4* to investigate the contribution of angiogenesis to lung development, and determine whether impaired DLL4-dependent angiogenesis disrupts alveolarization. Our data suggest that *Dll4* haploinsufficiency programs deviant angiogenesis and altered alveolar type I cell development, resulting in dysmorphic vascularization and disrupted alveolarization.

## Results

### DLL4 is expressed during mouse and human lung ontogeny.

**Figure 1:**
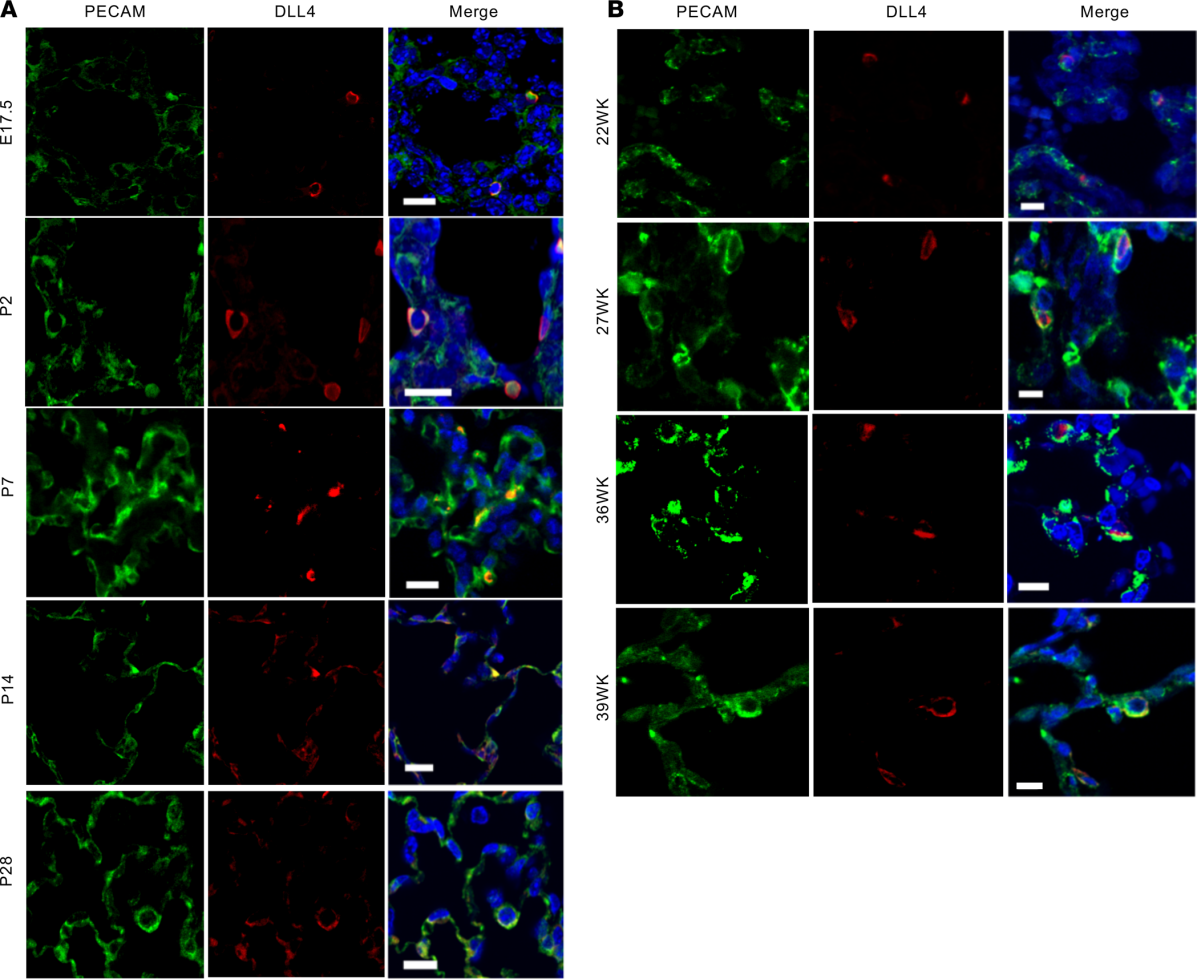
DLL4 is expressed from the canalicular through alveolar stage of mouse and human lung development. (**A**) DLL4 (red), PECAM (green), and nucleus (DAPI, blue) staining on mouse lung samples at E17.5, P2, P7, P14, and P28. E17.5 represents canalicular stage; P2 represents saccular stage; and P7, P14, and P28 represent early, middle, and completed alveolar stage. (**B**) DLL4 (red) and PECAM (green) staining on human lung slides at 22, 27, 36, and 39 gestational age weeks (22, 27, and 36/39 week represent human canalicular, saccular, and early alveolar stages). Scale bars: 10 μm. *n* = 4 per group.

**Figure 2:**
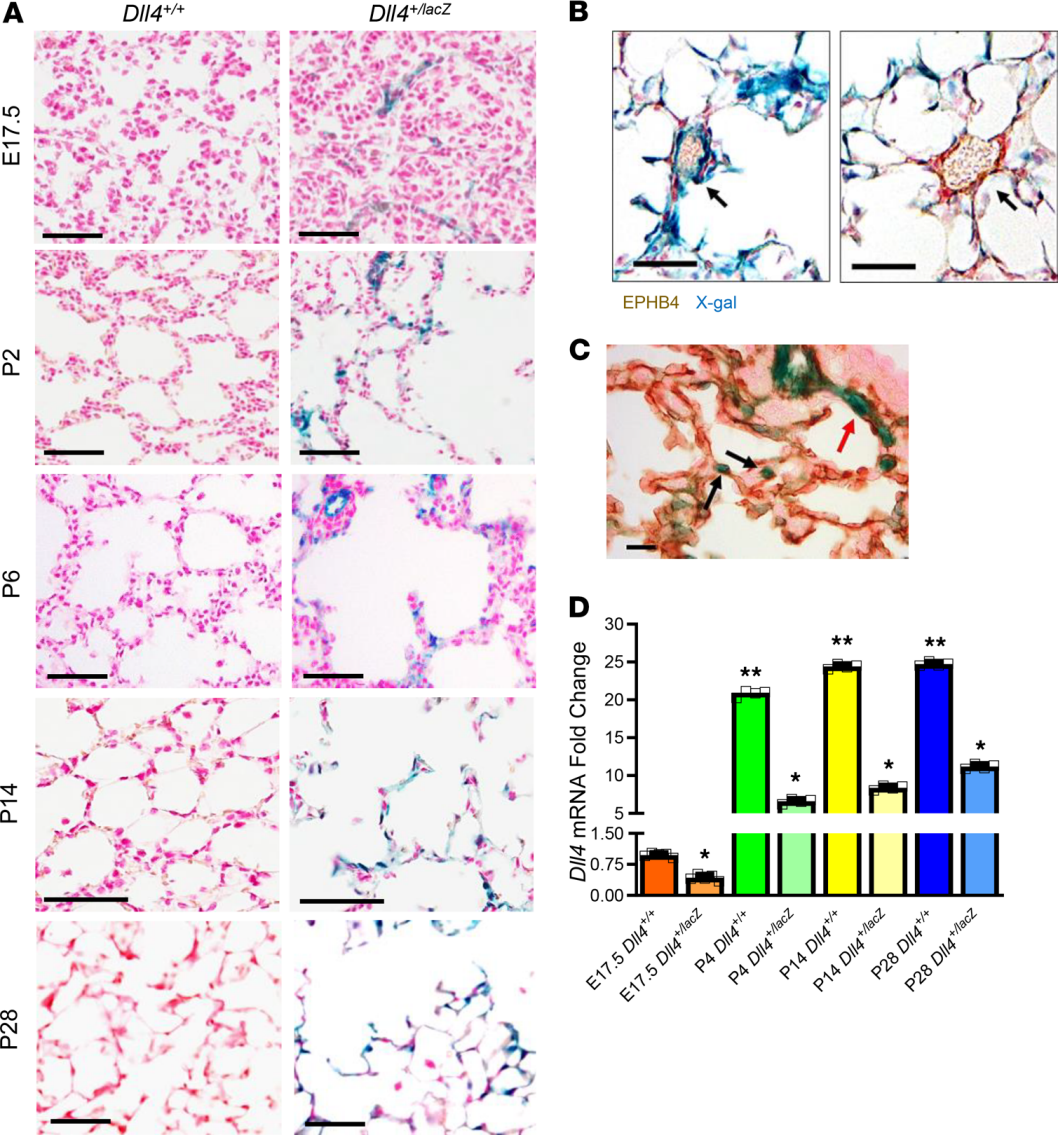
DLL4 lung mapping using the *Dll4^+/lacZ^* reporter mice. (**A**) X-gal staining on *Dll4^+/+^* and *Dll4^+/lacZ^* mouse lungs. Blue color represents *lacZ* gene expression, which indicates DLL4 expression, and fast red (red) represents nucleus (*n* = 3). *Dll4^+/+^* samples were used as X-gal negative control. (**B**) X-gal and EPHB4 IHC (brown) staining on P14 mouse sample shows that DLL4 is not expressed in EPHB4^+^ cells. The arrow (left panel) points to an intermediate Dll4^+^ EPHB4**^–^** blood vessel, and the arrow (right panel) points to a Dll4**^–^** EPHB4^+^ blood vessel. (**C**) X-gal and PECAM IHC (brown) staining on P6 mouse lung sample. The black arrows point to the leading EC of blood vessel, and the red arrow points to a capillary branching from a small artery. (**D**) qRT-PCR showing mouse whole lung *Dll4* expression from the canalicular to alveolar stage in WT and *Dll4^+/lacZ^* mice. *n* = 5 mice per group; *P < 0.01, E17.5 *DLL4^+/+^* vs. P4, P14, P28 *DLL4^+/+^* mice; **P < 0.01, E17.5 *DLL4^+/lacZ^* versus P4, P14, P28 *DLL4^+/lacZ^* mice. Scale bars: 50 μm (**A** and **B**); 10 μm (**C**). Data are shown as mean ± SD throughout. (**D**) One-way ANOVA with Tukey’s test.

To determine whether DLL4 is important for lung vascularization we mapped DLL4 in canalicular (C57BL/6 mouse [M] — E17.5, human [H] — 22 weeks gestational age [GA]), saccular (M: P2; H: 27-week GA), and alveolar (M: P7, P14, and P21; H: 36- and 39-week GA) stages in mouse (C57BL/6) and human autopsy samples. In the human and mouse lungs, DLL4 (red) was expressed in a PECAM-restricted manner (green) from the canalicular phase (Figure 1, A and B). DLL4 expression was scant in the canalicular phase, increased in the saccular phase (P2), and was widely distributed in the early, middle, and late alveolar phase (Figure 1A). Similar patterns of DLL4 expression were found in cadaveric human lung sections obtained from the late canalicular (22 week), saccular (27 week), and early alveolar (36 and 39 week) stages (Figure 1B). To confirm DLL4 antibody staining, we performed X-gal staining on frozen lung sections of *Dll4^+/lacZ^* CD1 pups, where 1 *Dll4* allele is replaced by the *lacZ* reporter (11). LacZ expression was sparse in the canalicular stage, but showed abundant expression during postnatal lung development (Figure 2A), consistent with our mapping studies in C57BL/6 mice and human autopsy specimens. Costaining X-gal (blue) with EPHB4 (brown, Figure 2B) and PECAM (brown, Figure 2C), respectively, showed that *Dll4* is expressed in an EC–restricted manner in branching capillaries and arteries, but not in veins (Figure 2, B and C).

We next quantified temporal changes in *Dll4* RNA in mouse lungs at E17.5, P4, P14, and P28 to determine whether postnatal increase in pulmonary microvascular arborization coincides with changes in *Dll4* expression. *Dll4* mRNA expression increased exponentially from canalicular stage to saccular phase and remained elevated through the alveolarization stage (Figure 2D), with 50% reduction in *Dll4^+/lacZ^* mice (Figure 2D). During angiogenesis, EC tip cells, enriched in DLL4, lead sprouting angiogenesis, and stalk cells follow the tip cell. DLL4 expression in the leading EC was observed with X-gal/PECAM IHC staining in *Dll4^+/lacZ^* mouse lungs (Figure 2C), and with DLL4/PECAM immunofluorescent (IF) staining in human and C57BL/6 mouse lung samples (see below). Our data from human samples and from 2 mouse strains (CD1, C57BL/6) demonstrate that *Dll4* is expressed during lung ontogeny from the canalicular phase, supporting its role in pulmonary vascular development.

### Dll4 predominantly expresses in lung EC.

**Figure 3:**
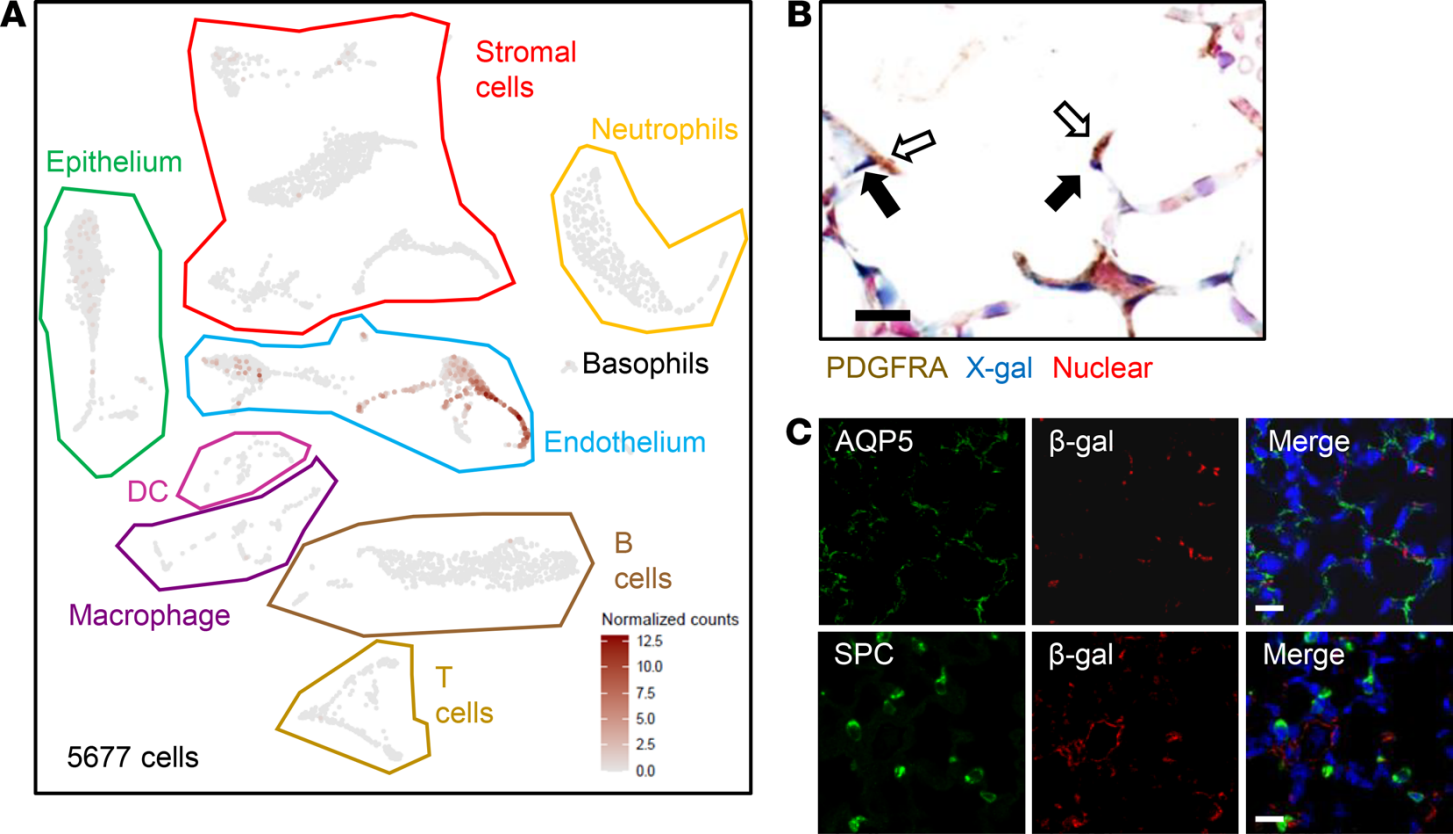
DLL4 prominently expresses in EC. (**A**) Three C57BL/6 WT mouse lungs were collected on P14 for single-cell RNA-Seq, and *Dll4* expression plot was generated from 5677 cells. Epithelium, endothelium (EC), and stromal cells were bordered by green, blue, and red lines separately. Note: DLL4 (brown dots) expression is predominantly in the EC cluster. (**B**) *Dll4^+/lacZ^* mouse lungs were harvested and cryosections were obtained on P14, and then X-gal staining and PDGFRA IHC staining were performed. Blue, brown, and red colors indicate X-gal, PDGFRA, and nuclear staining, respectively. Hollow arrows point to PDGFRA^+^ cells, and solid arrows point to X-gal^+^ cells. (**C**) Mouse lungs were harvested from 14-day-old *Dll4^+/lacZ^* mice. AQP5 (green) and SPC (green) (IF) were costained with β-galactosidase (red) separately. *n* = 5 per group. Scale bars: 10 μm.

To confirm that *Dll4* expression is predominantly restricted to EC in the lung, we applied single-cell RNA-Seq (scRNA-Seq) in P14 C57BL/6 mouse lungs (*n* = 3). We identified 4 major groups of cells in 5677 cells pooled from 3 mice using established marker genes (19): epithelial cells (*Epcam* and *Cdh1*), ECs (*Pecam* and *Cdh5*), stromal cells (*Col1a1* and *col1a2*), and immune cells (*Ptprc*), which included DCs, macrophages, B cells, T cells, basophils, and neutrophils (Figure 3A). We next generated *Dll4* expression plots, which showed that *Dll4* is mainly expressed in EC (Figure 3A). To verify scRNA-Seq results, we stained PECAM (EC marker), PDGFRA (myofibroblast marker), AQP5 (AT1 marker), and SPC (AT2 marker) with X-gal or anti–β-gal costaining separately. X-gal and PDGFRA IHC staining indicated that PDGFRA^+^ fibroblasts did not express *Dll4* (Figure 3B). Additionally, β-gal and AQP5/SPC IF staining showed that *Dll4* did not colocalize with AT1 and AT2 cells (Figure 3C). However, DLL4/PECAM IF staining (Figure 1, A and B) and X-gal/PECAM IHC staining (Figure 2C) revealed that *Dll4* expressed in EC. Our scRNA-Seq and immunostaining data demonstrate that *Dll4* is prominently expressed in pulmonary EC.

### Altered expression of EC genes that specify EC fate in Dll4^+/lacZ^ mice.

**Figure 4:**
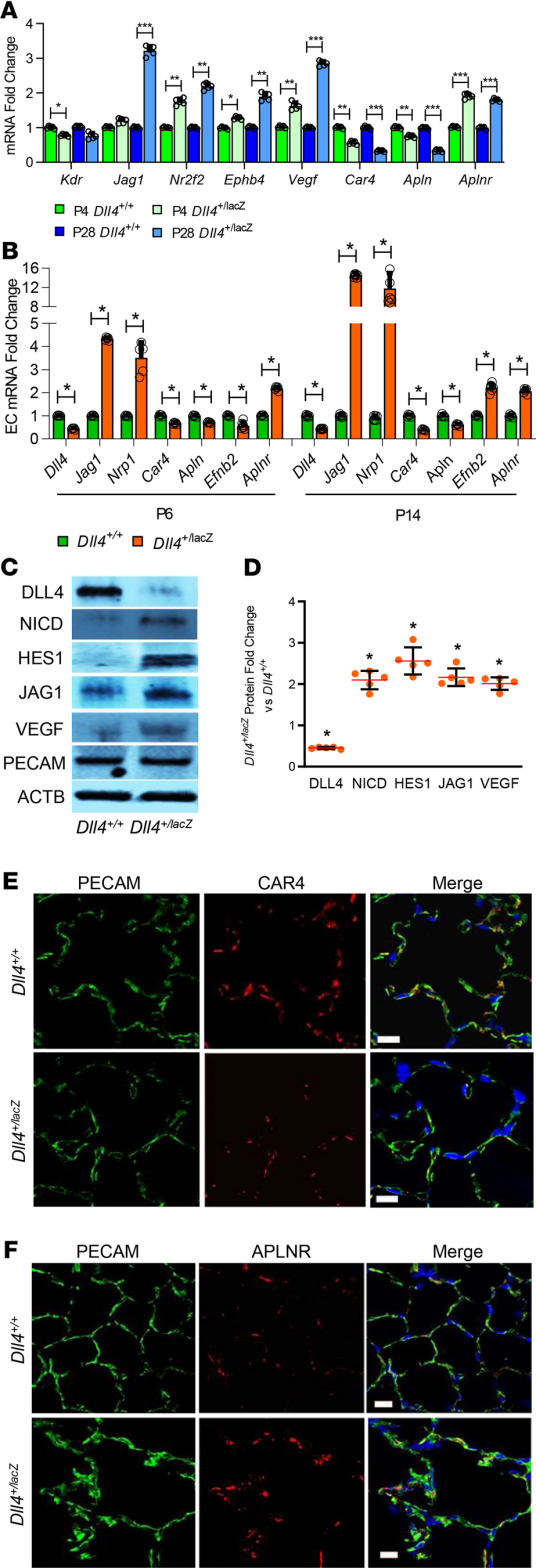
Altered EC gene expression in the ECs of *Dll4^+/lacZ^* mice. (**A**) Whole-lung RNA homogenates obtained from *Dll4^+/+^* and *Dll4^+/lacZ^* at P4 and P28 were used to quantify *Kdr, Jag1, Nr2f2, Ephb4, Vegfa, Car4, Esm1, Apln,* and *Aplnr* with qRT-PCR. *n* = 5 mice per group; *P < 0.05, **P < 0.01, ***P < 0.001. (**B**) Mouse lung ECs (PECAM pulldown) obtained from *Dll4^+/+^* and *Dll4^+/lacZ^* at P6 and P14 were used to quantify *Dll4, Nrp1, Jag1, Car4, Apln, Esm1, Efnb2, and Aplnr*. *n* = 5 mice per group; *P < 0.01. (**C**) Mouse lung ECs were harvested from 14-day-old *Dll4^+/+^* and *Dll4^+/lacZ^* mice. Lung homogenates were used to quantify DLL4, NICD, HES1, JAG1, VEGFA, and PECAM by immunoblotting, with densitometry shown graphically (**D**). *n* = 5 mice per group; *P < 0.01. (**E**) PECAM (green), CAR4 (red), and DAPI staining on P14 *Dll4^+/+^* and *Dll4^+/lacZ^* lung sections (*n* = 4). (**F**) PECAM (green), APLNR (red) and DAPI (blue) staining on *Dll4^+/+^* and *Dll4^+/lacZ^* mouse lung slides (*n* = 4). Scale bars: 10 μm. Data are shown as mean ± SD. (**A**, **B**, and **D**, Gaussian distribution used 1-way ANOVA with Tukey’s test) (**A** and **B**) Non-Gaussian distribution used 2-tailed Mann-Whitney *U* test.

We compared gene expression that specifies EC fate during sprouting angiogenesis between *Dll4^+/+^* and *Dll4^+/lacZ^* mice. We focused on VEGFA and VEGF receptor 2 (KDR) in VEGF/KDR pathway, DLL4, JAG1, Notch intracellular domain (NICD), and HES1 in Notch pathway. A recent study identified that *Car4* and *Apln* mark alveolar capillary EC that resemble tip cells, whereas *Aplnr* mark alveolar capillary EC that resemble stalk cells (20). VEGFA stimulates endothelial angiogenesis and regulates EC survival (21). We noted that *Kdr* expression decreased at P4, whereas *Nr2f2* (venous EC marker) and *Vegfa* expression increased in the whole lung at P4 and P28 in *Dll4^+/lacZ^* mice (Figure 4A). We also noted increased Nrp1 expression, the Kdr coreceptor in lung EC of *Dll4^+/lacZ^* mice (Figure 4B). We confirmed that VEGFA protein was increased in lungs of *Dll4^+/lacZ^* mice by Western blotting (Figure 4, C and D). These data suggest that *Dll4* haploinsufficiency programs decreased *Kdr* expression, while inducing compensatory upregulation of *Vegfa*. *Jag1* RNA and protein expression was increased in mouse whole lung (P28) and lung EC (P6, P14) during postnatal lung development (Figure 4, A–D). Expression of NICD, a cleavage product of Notch1, and HES1 was induced strongly in *Dll4^+/lacZ^* mouse EC (Figure 4, C and D) in relation with increased JAG1 but decreased DLL4. In lung, CAR4^+^/APLN^+^ EC locate next to AT1 cells and APLNR^+^ EC localize to the side away from the air space (20). There was less *Car4* and *Apln* expression and more *Aplnr* expression in mouse whole lung (P4 and P28) and pulmonary EC (P6 and P14) (Figure 4, A and B). PECAM costaining with CAR4 or APLNR confirmed less CAR4^+^ EC and more ARLPN^+^ EC in *Dll4^+/lacZ^* mouse lung compared with *Dll4^+/+^* mouse lung (Figure 4, E and F). We also found that *Ephb4* mRNA expression was increased in whole lungs at P4 and P28,and that *Efnb2* mRNA expression was decreased at P6 but increased at P28 in pulmonary EC (Figure 4, A and B). These data indicate polarization of lung EC toward a JAG1/NICD/HES1 fate in *Dll4^+/lacZ^* mice, and the lung capillary EC population shifts to an *Aplnr^+^* expression signature from a *Car4^+^/Apln^+^* signature.

### Dll4 haploinsufficiency causes defective vascular network in the developing lung.

**Figure 5:**
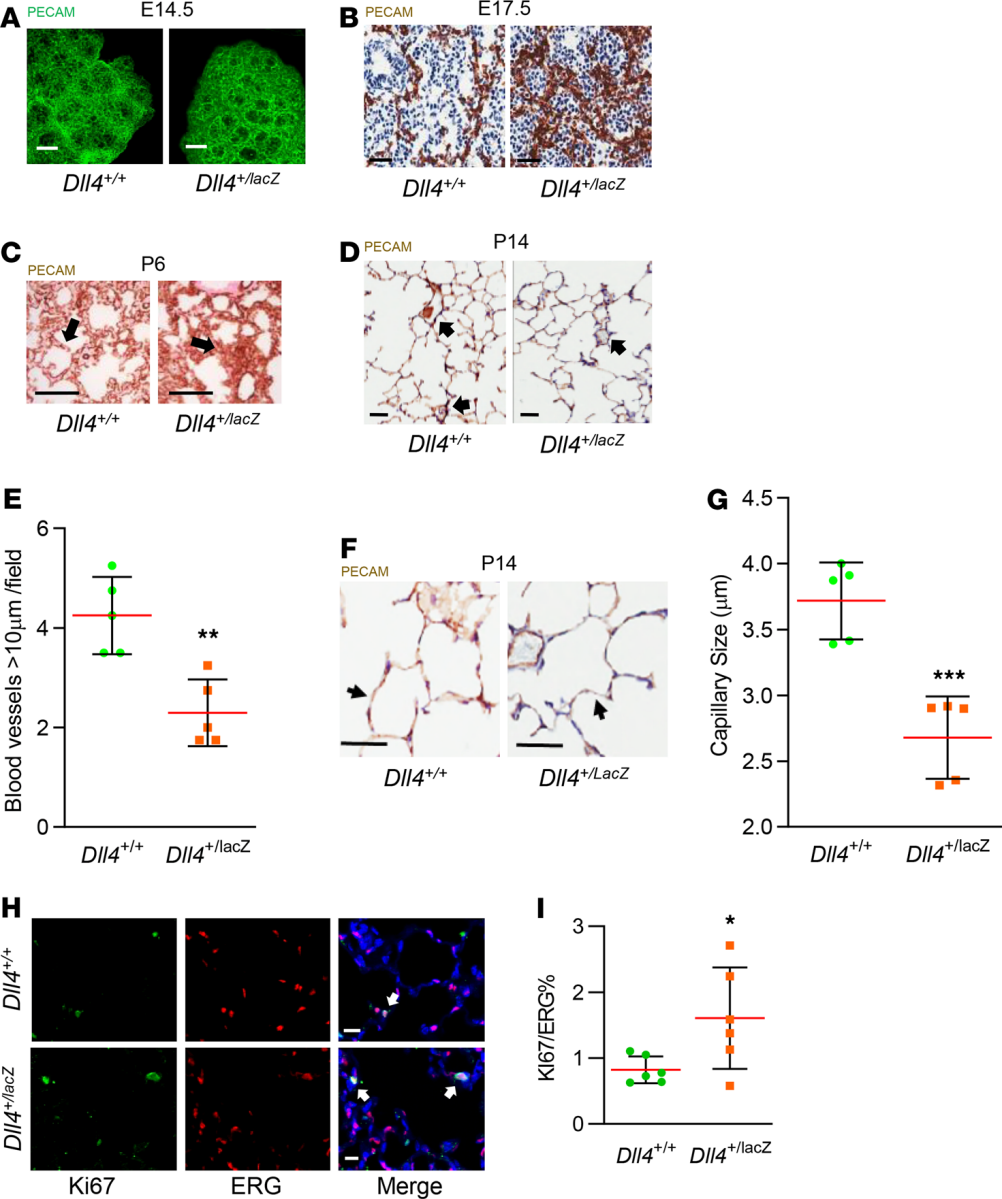
Dysmorphic vascular development in *Dll4* mutant mice. (**A**) PECAM (green) whole-mount staining on E14.5 *Dll4^+/+^* and *Dll4^+/lacZ^* mouse lungs, *n* = 4 per group. (**B**) PECAM (brown) and Harris Hematoxylin (H) (blue) staining on E17.5 *Dll4^+/+^* and *Dll4^+/lacZ^* mouse lungs. PECAM staining indicates much denser vascular structure in *Dll4^+/lacZ^* mouse lung at canalicular stage. Note: Lack of bronchioles in *Dll4^+/lacZ^* lung, *n* = 5 per group. (**C**) PECAM (brown) and fast red (red) staining on P6 *Dll4^+/+^* and *Dll4^+/lacZ^* mouse lungs. A double capillary network was visualized in *Dll4^+/+^* mice, but “whorls” of misaligned network were found in *Dll4^+/lacZ^* mouse lung at early alveolar stage, *n* = 4 per group. The arrow points to the double capillary in the left panel, and the arrow points to the misaligned network in the right panel. (**D** and **F**) PECAM (brown) and H staining on P14 *Dll4^+/+^* and *Dll4^+/lacZ^* mouse lungs. The arrows in (**D**) point to intermediate blood vessels, and the arrows in (**F**) point to the capillaries, with quantifications shown for intermediate blood vessel number (**E**) and capillary thickness (**G**), which were less at P14 in *Dll4^+/lacZ^* mouse lung. *n* = 5 mice per group, **P < 0.01, ***P < 0.001. (**H**) Ki67 (green), ERG (red), and DAPI (blue) staining on P14 mouse lung slides, with quantifications shown for Ki67^+^ cells percentage in total ERG^+^ cells (**I**). *n* = 6 mice per group, *P* < 0.05. The arrows in (**H**) point to Ki67^+^ERG^+^ cells. Scale bars: 100 μm (**A**); 200 μm (**B**); 100 μm (**C**); 25 μm (**D** and **F**); 10 μm (**H**). Data are shown as mean ± SD. (**D**, **G**, and **F**) One-way ANOVA with Tukey’s test.

To investigate the effect of *Dll4* haploinsufficiency on lung vascular arborization, we performed PECAM mapping. PECAM whole-mount lung staining at E14.5 revealed subtle increase in vascular loops in *Dll4^+/lacZ^* mice with denser networks (Figure 5A). Additionally, we noted exuberant, misaligned capillary growth in *Dll4^+/lacZ^* mice with fewer bronchioles at E17.5 (Figure 5B). At P6, a double capillary network was visualized in *Dll4^+/+^* mice; however, in *Dll4^+/lacZ^* mice we found “whorls” of misaligned, dysmorphic capillary networks (Figure 5C). We posited that deviant capillary arborization will impair microvascularization in the alveolar phase. At P14, the number of blood vessels ranging in size 10–150 μm decreased by 50% (Figure 5, D and E), whereas the average capillary diameter reduced from 3.8 to 2.7 μm (*P* < 0.001) in *Dll4^+/lacZ^* mice (Figure 5, F and G). To verify whether *Dll4* haploinsufficiency influences EC proliferation, Ki67 and ERG double IF staining was performed on P14 mouse lung sections, which revealed more EC proliferation in *Dll4^+/lacZ^* lung EC (Figure 5, H and I). However, we did not observe significant differences in lung EC apoptosis with ERG and TUNNEL double staining between *Dll4^+/+^* and *Dll4^+/LacZ^* mice at P14 (data not shown). These data demonstrate that *Dll4* heterozygosity stimulates EC proliferation and programs dysmorphic vascular patterning characterized by hypervascular, misaligned vessels in the canalicular and saccular stages, and decreased capillary caliber and number of small pulmonary blood vessels in the alveolar stage.

### Dysmorphic vascular development in Dll4^+/lacZ^ mice impairs alveolarization.

**Figure 6:**
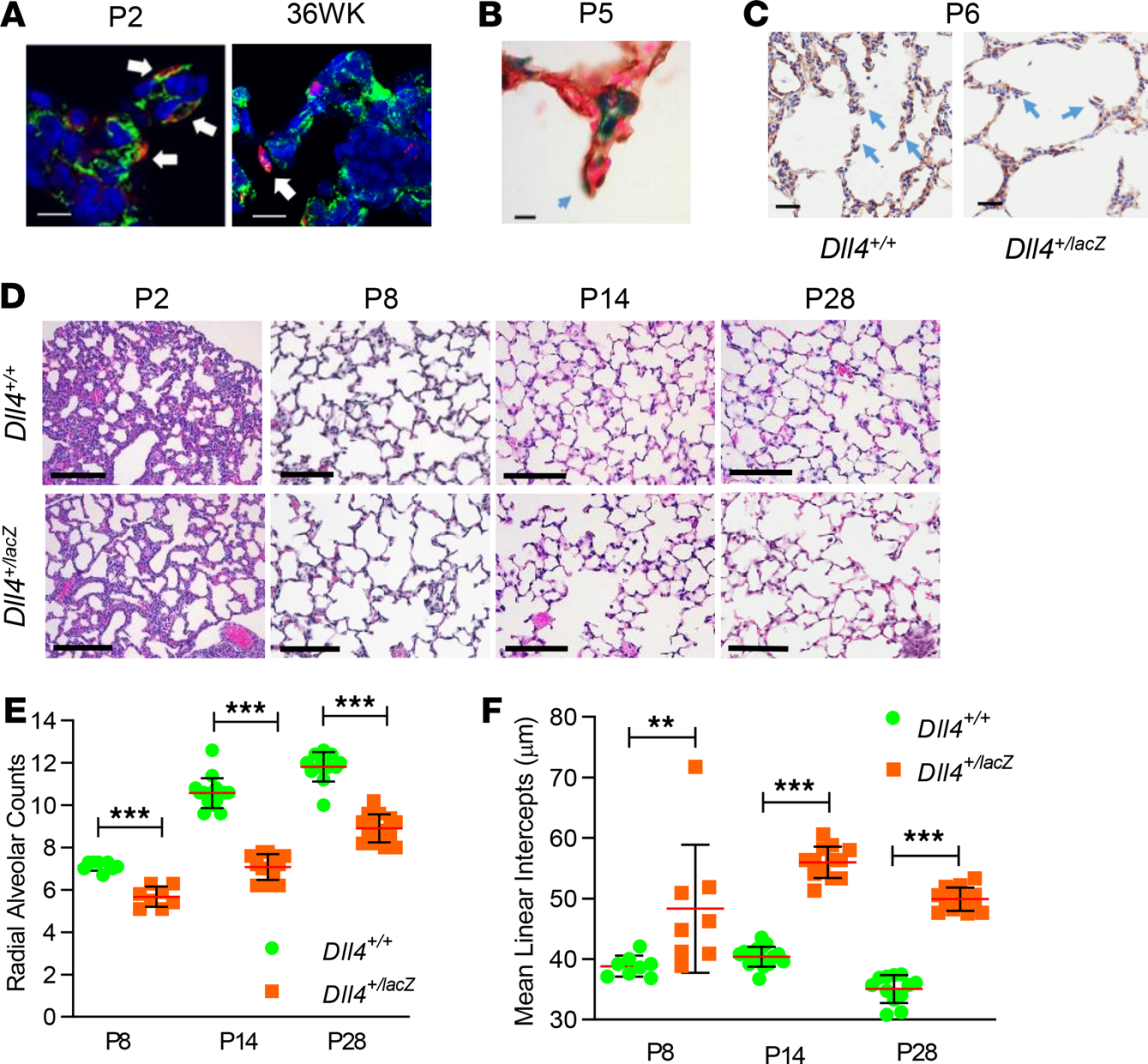
*Dll4* haploinsufficiency causes defective alveolarization. (**A**) DLL4 (red), PECAM (green), and DAPI (blue) staining indicates DLL4 is expressed in the leading EC localized in primary septa at P2 in mouse lung (left panel) and at 36 weeks in human lung (right panel). The arrows point to the DLL4^+^ leading EC. (**B**) X-gal (blue) and PECAM IHC (brown) staining shows that DLL4 is expressed in the leading EC localized in primary septa at P6 in mouse lung. The arrow points to the DLL4^+^ leading EC. (**C**) PECAM IHC (brown) and Harris (blue) staining in *Dll4^+/+^* and *Dll4^+/lacZ^* P6 mouse lung showing blunt septa in *Dll4^+/lacZ^* compared with *Dll4^+/+^*. The arrows point to normal septa in *Dll4^+/+^* in the left panel and blunt septa in *Dll4^+/lacZ^* in the right panel. (**D**) H&E staining on P2, P8, P14, and P28 *Dll4^+/+^* and *Dll4^+/lacZ^* mouse lungs. Radial alveolar counts (RAC) (**E**) and mean linear intercepts (MLI) (**F**) at P8, P14, and P28 shown graphically. *n* = 5 mice/group, **P < 0.01, ***P < 0.005. Scale bars: 10 μm (**A** and **B**); 25 μm (**C**); 100 μm (**E**). Data are shown as mean ± SD. (**F**) Two-tailed Mann-Whitney *U* test.

DLL4/PECAM IF staining revealed DLL4 is expressed in the leading EC of capillaries in alveolar septa of human and mouse lungs (Figure 6A), with X-gal/PECAM IHC staining confirming DLL4 in the alveolar septum of *Dll4^+/lacZ^* mice (Figure 6B). We posited that deviant vascular patterning seen with *Dll4* haploinsufficiency will impact alveolar septal development and alveolarization. As early as the canalicular stage, we noted that terminal bronchiole development was impaired in *Dll4^+/lacZ^* mice (Figure 5B). Blunt primary and underdeveloped secondary septa were observed at P6 in *Dll4^+/lacZ^* mouse lungs (Figure 6C). Lung morphology was clearly altered by the saccular stage (P2) (Figure 6D), and lung morphometry during the early and mid-alveolar phase (P8 and P14) as well as in the mature lung (P28) revealed decreased radial alveolar counts and increased mean linear intercepts in *Dll4^+/lacZ^* mice compared with *Dll4^+/+^* littermates (Figure 6, D–F). Impaired alveolarization in *Dll4^+/lacZ^* mice was observed in both male and female pups. Although it is not technically feasible to inflate P2 lungs, and alveoli are not formed, we did assess the number of completed air sacs distal to the respiratory bronchioles. We found a 25% reduction in the number of distal air spaces (*Dll4^+/lacZ^* vs. *Dll4^+/+^*; 1.7 ± 0.5 vs. 2.4 ± 0.4 SD, *P* < 0.02, *n* = 9 and 5, respectively). These data demonstrate that DLL4 is required for lung alveolarization, and loss of 1 *Dll4* copy disrupts distal lung morphogenesis.

**Figure 7:**
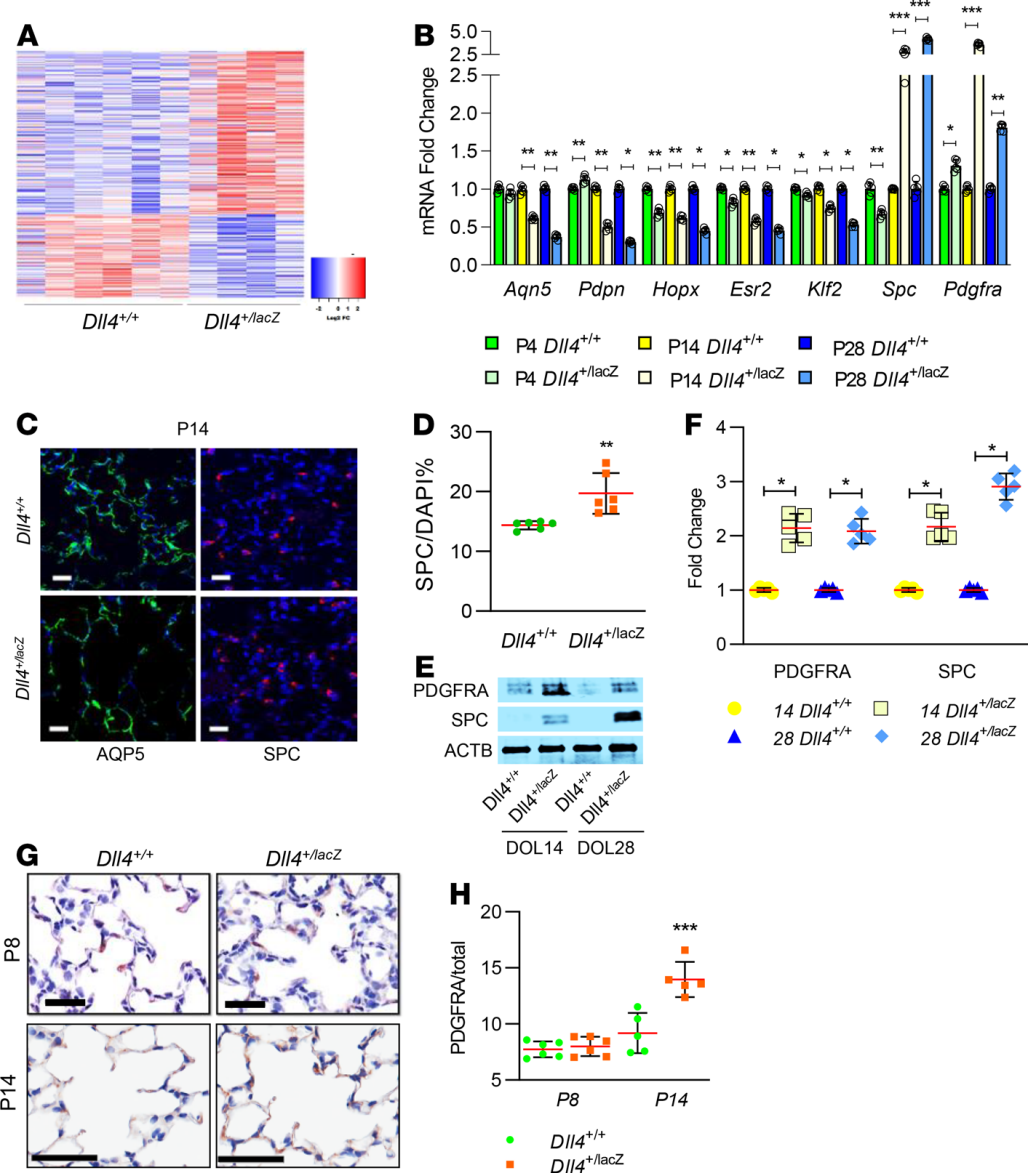
RNA-Seq identifies lung genes associated with deviant vascular and alveolar development with *Dll4* haploinsufficiency. (**A**) The right lungs from *Dll4^+/+^* (*n* = 6) and *Dll4^+/lacZ^* (*n* = 4) mice were harvested on P6, and RNA extracted was used for RNA-Seq using an Illumina NovaSeq platform. Heatmap of 336 differentially expressed genes between *Dll4^+/+^* (*n* = 4) and *Dll4^+/lacZ^* (*n* = 4) mouse lungs (*P* < 0.05). (**B**) qRT-PCR demonstrates *Aqp5*, *Pdpn*, *Hopx,* *Esr2*, *Klf2, Spc,* and *Pdgfr*α expression in *Dll4^+/+^* and *Dll4^+/lacZ^* mouse lungs at P4, P14, and P28. (**C**) AQP5 (green) and SPC (red) IF staining with DAPI (blue) in *Dll4^+/+^* and *Dll4^+/lacZ^* mouse lungs at P14, with quantification shown for percentage of SPC^+^ cells of the total DAPI-stained cells (**D**), *n* = 6 in each group and *P* < 0.005. (**E**) Mouse lungs were harvested from *Dll4^+/+^* and *Dll4^+/lacZ^* mice at P14 and P28. Lung homogenates were used to quantify SPC and PDGFRA by immunoblotting, with densitometry shown graphically (**F**), *n* = 5 mice per group; *P* < 0.01. (**G**) PDGFRA (brown) IHC staining with Harris staining (blue) on P8 and P14 *Dll4^+/+^* and *Dll4^+/lacZ^* mouse lung sections, with quantification shown for percentage of PDGFRA^+^ cells in total Harris-stained cells (**H**), *n* = 6 in each group, *P < 0.05, **P < 0.01, and ***P < 0.001. Scale bar: 20 μm. Data are shown as mean ± SD. (**B**, **F**, and **H**) Gaussian distribution used 1-way ANOVA with Tukey’s test. (**D**) Gaussian distribution with 1 comparison used 2-tailed *t* test with Welch’s correction.

To gain unbiased mechanistic insights underlying deviant vascular and alveolar development with *Dll4* haploinsufficiency, we performed whole lung RNA-Seq in *Dll4^+/+^* (*n* = 6) and *Dll4^+/lacZ^* (*n* = 4) at P6 (Figure 7A). The top 10 gene networks altered and major cardiovascular pathways are shown in Supplemental Table 1; supplemental material available online with this article; https://doi.org/10.1172/jci.insight.134170DS1 Gene networks regulating angiogenesis and vascular development were upregulated in *Dll4^+/lacZ^* lungs (*P* < 0.01; Supplemental Table 1). Specifically, genes such as *Foxn4* (fold change [FC] = 2.2, *P* = 0.03), *Thbs4* (FC = 3.3, *P* = 0.02)*, Lrg1* (FC = 4.0, *P* = 0.03)*,* and *Ccl2* (FC = 2.2, *P* = 0.04) implicated in pathological angiogenesis were upregulated, whereas genes known to suppress angiogenesis such as *Stat1* (FC = 0.59, *P* = 0.02)*, Il17F* (FC = 0.12, *P* = 0.049)*,* and *Klf2* (FC = 0.74, *P* = 0.02) were suppressed. We then performed exploratory analysis to determine whether the lack of *Dll4* altered maturation of alveolar type I (AT1) or alveolar type II (AT2) cells, key constituents of the mature alveoli. Ingenuity pathway analysis predicted delayed maturation of AT1 cells in *Dll4^+/lacZ^* mice based on decreased *Esr2* (FC = 0.32, *P* < 10^–4^) and *Klf2* (FC = 0.74, *P* < 0.02) expression. Validation of RNA-Seq data by quantitative reverse transcription PCR (qRT-PCR) in independent lung samples from *Dll4^+/+^* and *Dll4^+/lacZ^* mice confirmed progressive decrease in *Esr2* and *Klf2* expression on P4, P14, and P28 with *Dll4* deficiency (Figure 7B). We next quantified temporal changes in expression of AT1 lineage markers *Hopx, Aqpn5,* and *Pdpn* in *Dll4^+/lacZ^* and *Dll4^+/+^* littermates. *Hopx* expression was decreased on P4, but all AT1 markers were decreased on P14 and P28 in *Dll4^+/lacZ^* mice (Figure 7B). AQP5^+^ AT1 cells were continuously distributed along alveolar inner lining surface in *Dll4^+/+^* lungs, but showed an interrupted pattern in *Dll4^+/lacZ^* lungs (Figure 7C). SPC^+^ AT2 cells were increased in *Dll4^+/lacZ^* lungs at P15 (Figure 7, C and D). Immunoblotting and PCR confirmed there was more SPC expression in developing lungs at P14 and P28 (Figure 7, B, E, and F). PDGFRA^+^ fibroblasts interact with EC and alveolar epithelial cells, and secrete extracellular matrix molecules in the newly formed septa during lung alveolarization (22), and, therefore, we quantified PDGFRA expression and PDGFRA^+^ cells. We found increased PDGFRA RNA and protein expression at P14 and P28 (Figure 7, B, E, and F) and more PDGFRA^+^ cells at P14 with IHC staining (Figure 7, G and H) in *Dll4^+/lacZ^* lungs. These data imply *Dll4* deficiency programs aberrant AT1 development with increases in AT2 and PDGFRA^+^ fibroblasts that contribute to impaired lung alveolarization.

### Dll4 deficiency causes deviant angiogenesis in human lung ECs.

**Figure 8:**
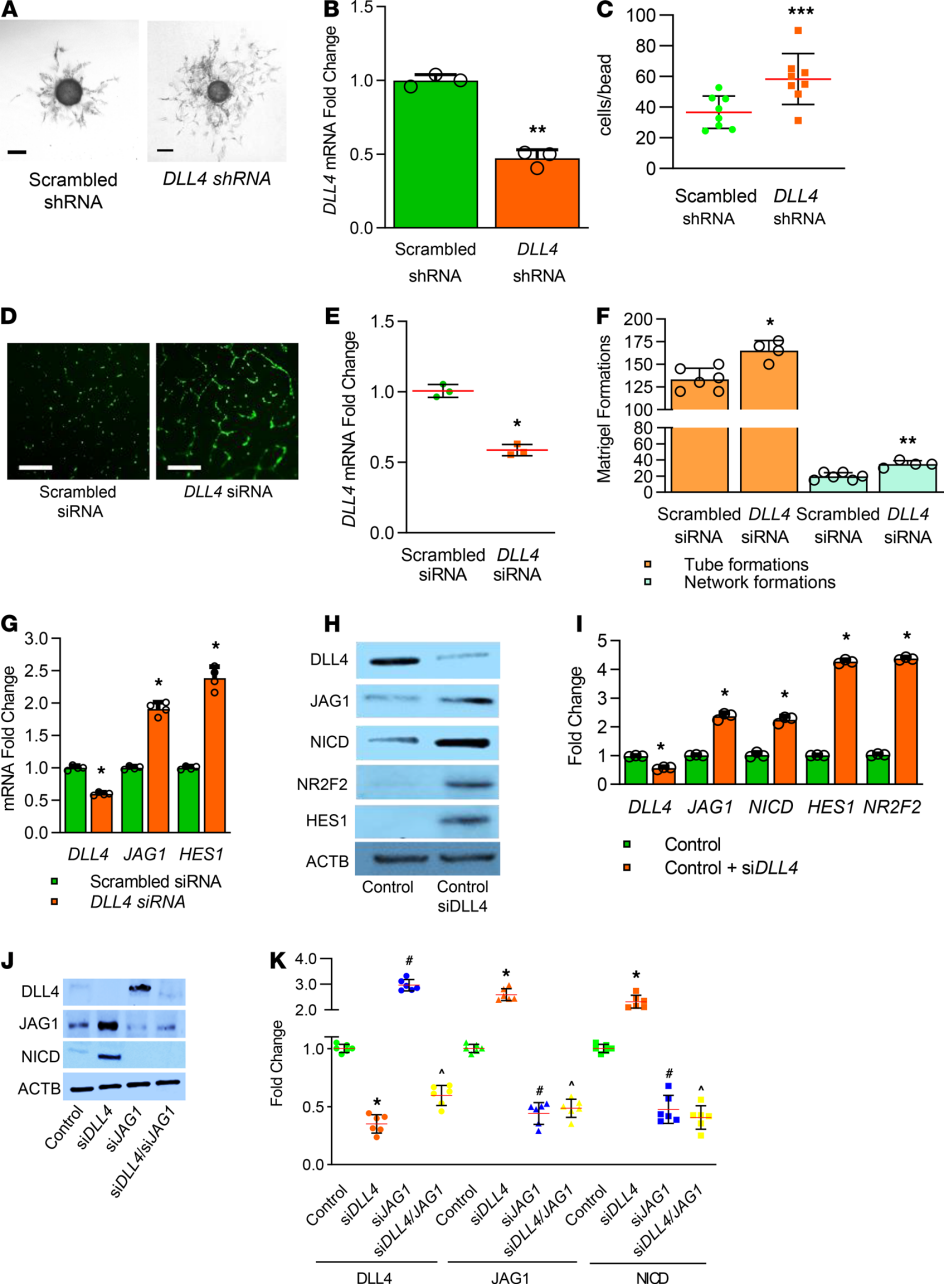
*DLL4* deficiency programs a hypersprouting angiogenic phenotype in HLEC in vitro. (**A**) *DLL4-*deficient HLEC-Im show hyperbranching network in 3D cell culture compared with control EC, with quantifications shown for average cell numbers per bead (**C**), 1–4 beads per well, 8 wells in both groups, *P* < 0.001. (**B**) qRT-PCR indicates that *Dll4* is knocked down around 50% in *DLL4-*deficient HLEC-Im. *n* = 3 per group, *P* < 0.01. (**D**) *DLL4* partially silenced in primary HLEC using si*DLL4* shows increased branching and network formation in Matrigel compared with EC treated with scrambled siRNA with quantifications shown graphically (**F**), *P < 0.02, **P < 0.01. (**E**) qRT-PCR indicates that si*DLL4* reduced *DLL4* in primary HLEC by around 50%. *N* = 4 per group, *P* < 0.03. (**G**) qRT-PCR from HLEC shows a 50% decrease with si*DLL4* and an increase in both *JAG1* and *HES1*. *n* = 4 per group; *P < 0.03, control vs. *siDLL4*. (**H**) HLEC were harvested after siRNA treatment and homogenates were used to quantify DLL4, JAG1, NICD, NR2F2, and HES1 by immunoblotting, with densitometry shown graphically (**I**). *n* = 3 per group; *P < 0.01, scrambled siRNA vs. si*DLL4*. (**J**) HLEC were harvested after scrambled siRNA, si*DLL4* or si*JAG1* treatment and homogenates were used to quantify DLL4, JAG1, and NICD by immunoblotting, with densitometry shown graphically (**K**). *n* = 5 per group; *P < 0.01, control vs. *siDLL4*; ^#^*P* < 0.01, control vs. *siJAG1*; ^*P* < 0.01, control vs. *siDLL4/siJAG1*. Scale bars: 250 μm (**A**); 100 μm (**C**). Data are shown as mean ± SD. (**C** and **K**) Gaussian distribution used 1-way ANOVA with Tukey’s test) (**B**, **E**, **F**, and **I**) Gaussian distribution with 1 comparison used 2-tailed *t* test with Welch’s correction. (**G**) Non-Gaussian distribution used 2-tailed Mann-Whitney *U* test.

We next investigated whether *DLL4* deficiency programs deviant angiogenesis in human lung ECs (HLECs). As primary HLECs do not survive 3D-angiogenesis assays, we immortalized primary HLEC (HLEC-Im) SV40 transformation. We then knocked down *DLL4* expression using lentivirus containing *DLL4* shRNA and mCherry in HLEC-Im. EC phenotype of HLEC-Im was confirmed with ERG and CDH5 staining, and AcLDL uptake assay (Supplemental Figure 1A). We sorted cells into 4 groups based on mCherry intensity (Supplemental Figure 1B), and then verified that medium mCherry intensity cells had about 50% *Dll4* knockdown (Figure 8B). 3D angiogenesis assays demonstrated that *DLL4* deficiency caused more EC proliferation, hyberbranching, dysmorphic and nondirectional angiogenesis in HLEC-Im (Figure 8, A and C).

To confirm 3D angiogenesis data in primary fetal HLEC, we used *DLL4* siRNA to reduce DLL4 expression by 50% (Figure 8E). *DLL4*-deficient HLEC had increased tube and network formation in 2D Matrigel assays (Figure 8, D and F). In *Dll4^+/lacZ^* mice, we had noted upregulation of JAG1-NICD signaling with DLL4 deficiency. To determine this signature shift is relevant in HLEC, and is cell autonomous, we quantified *JAG1* and *HES1* mRNA expression and found their expression increased with *DLL4* deficiency (Figure 8G). Silencing *DLL4* induced protein expression of JAG1, HES1, and NICD in parallel with increased expression of the venous marker, NR2F2 (Figure 8, H and I). To study whether upregulated NICD in *DLL4-*deficient EC is induced by increased JAG1, we knocked down *DLL4* and *JAG1* with siRNA separately and simultaneously. NICD expression was increased in *DLL4-*deficient EC, but decreased in *JAG1-*deficient EC (Figure 8, J and K). Interestingly, NICD expression was repressed in *DLL4* and *JAG1* double knockdown EC (Figure 8, J and K). These data confirm that *DLL4* deficiency cell autonomously induces JAG1-NICD signaling and deviant hypersprouting angiogenesis phenotype in human EC.

### Hyperoxia represses VEGF and DLL4 expression in the mouse lung and HLEC.

To investigate the relevance of DLL4 in neonatal lung disease, we used a neonatal model of hyperoxic lung injury in vivo and in vitro. We observed that VEGF and DLL4 protein and RNA expression in mouse lung was repressed by 3 days of 85% O_2_ treatment from P3 to P6 (Supplemental Figure 2, A–C). We also confirmed that VEGF and DLL4 protein expression in primary HLEC was repressed by 2 days of 85% O_2_ treatment (Supplemental Figure 2, D and E). These data showed that hyperoxia, a major risk factor for BPD, reduces DLL4 and VEGF expression in HLEC and mouse lungs.

## Discussion

**Figure 9:**
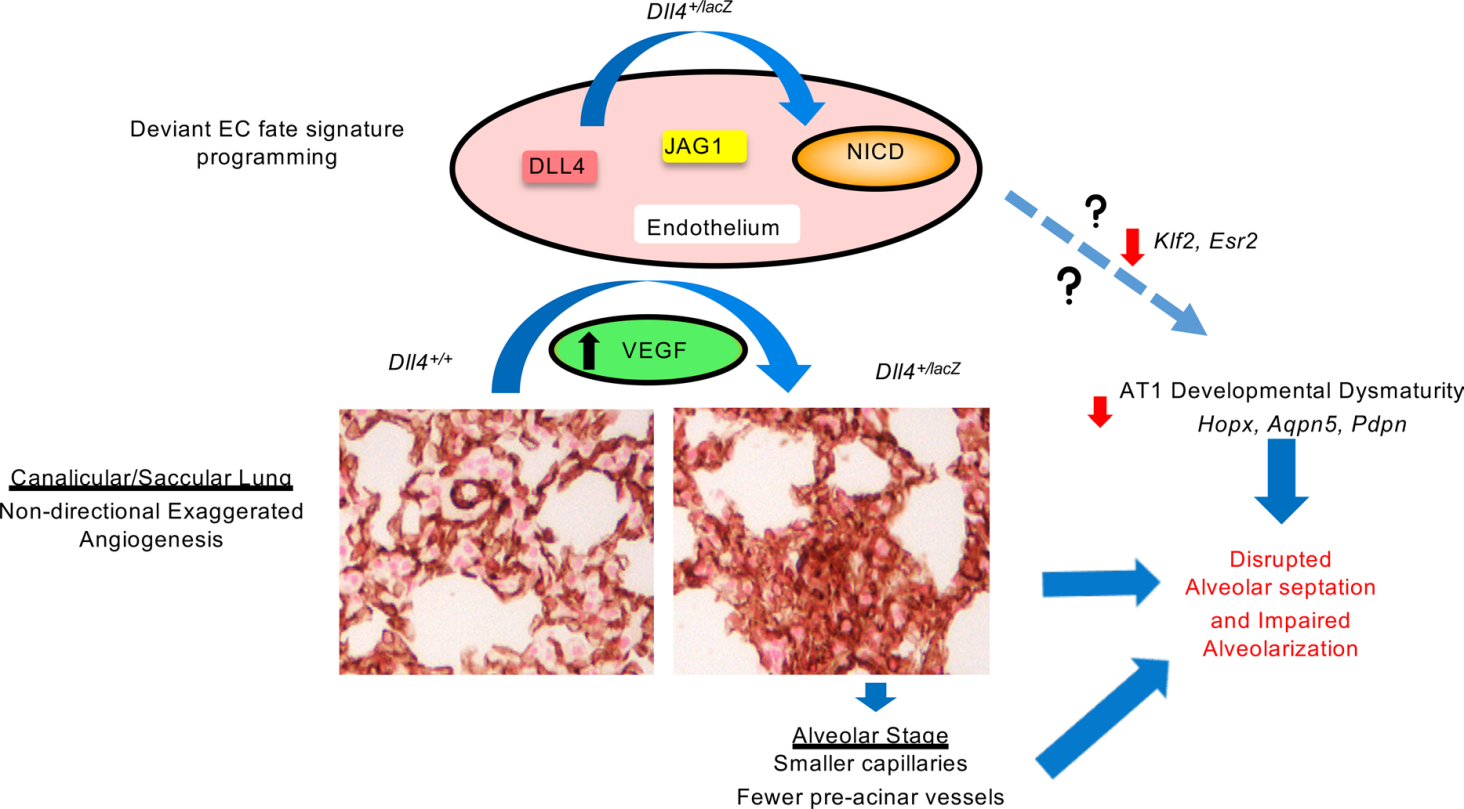
Illustration demonstrating mechanisms by which *DLL4* haploinsufficiency programs altered lung vascular and alveolar development in mice. AQP5, aquaporin 5; AT1, alveolar type I cell; DLL4, delta-like 4; Esr2, estrogen receptor 2; HOPX, homeodomain-only protein homeobox; JAG1, Jagged 1; Klf2, Kruppel-like factor 2; NICD, Notch intracytoplasmic domain; PDPN, podoplanin; VEGF, vascular endothelial growth factor.

The functional role of sprouting angiogenesis in distal lung microvascular arborization and alveolarization remains uncharacterized (1, 8, 9). Using mapping strategies, we show that DLL4 is expressed in both human and mouse lung from the canalicular through the alveolar stage of lung development. *Dll4* haploinsufficiency results in deviant lung microvascularization with an early “hypersprouting” angiogenic phenotype, followed by smaller capillaries and fewer distal vessels. These phenotypic changes occurred in parallel with skewing of lung EC expression signature toward JAG1/NICD activation and venous EC specification. Induced *DLL4* deficiency in human fetal lung EC autonomously programmed exaggerated angiogenic sprouting in vitro, reproducing an exaggerated JAG1/NICD activation signature. Strikingly, we noted that *Dll4* deficiency impaired distal lung acinar development from the canalicular stage with suppression of AT1 lineage markers (Figure 9). These data reveal an obligate role for DLL4 in pulmonary microvascular growth, arborization, and alveolarization.

Mapping strategies in C57BL/6, *Dll4^+/lacZ^* reporter mice, and human autopsy samples revealed signatures of DLL4 expression from the canalicular stage, with exponential increases from the saccular through alveolar stage. DLL4 expression was found in EC lining of lung arteries and in capillaries, but not veins, mimicking embryonal patterns (12, 23). DLL4 expression did not colocalize with AT1, AT2, or PDGFRA^+^ cells. scRNA-Seq confirmed that *Dll4* is predominantly expressed in the lung EC cluster on P14, consistent with similar distribution patterns during other stages of lung development in LungMap. We found DLL4 in the leading cells of PECAM^+^ staining capillaries, indicating sprouting angiogenesis (24, 25). Interestingly, temporal increase in DLL4 expression in the saccular and alveolar phase coincides with the rapid increase in lung microvascular growth postnatally. These mapping studies suggest a role for DLL4-regulated angiogenesis during distal lung ontogeny. Prior work by DeMello et al. based on morphological analysis of lung vascular casts suggested that vasculogenesis was critical for distal lung vascular development (6). Parera et al. mapped TIE2 (an EC receptor for angiopoietins) to suggest that angiogenesis contributes to distal lung vascularization (8). Use of lung vascular casts or mapping the ubiquitous EC receptor, TIE2, did not clarify the molecular mechanisms regulating distal lung vascularization, nor functionally examine the contribution of angiogenesis to alveolarization. Lung stage-specific mapping combined with morphological analysis clarifies the role of DLL4 in lung development.

*Dll4^–/–^* and *Dll4^+/–^* mice on the C57BL/6 background do not survive to the canalicular stage, dying by E11.5 of severe vascular defects. We therefore used *Dll4^+/lacZ^* mice on the CD1 background to study the impact of *Dll4* deficiency on lung development. We found that *Dll4* haploinsufficiency altered lung EC tip versus stalk cell behavior programming a hypersprouting and nondirectional capillary arborization pattern evident from the canalicular phase. This dysmorphic lung vascular patterning is similar to the hypersprouting, nonproductive angiogenesis phenotype described with *Dll4* deficiency during retinal vascularization (23, 26). In the retina, DLL4 represses tip cell division in *cis* and in *trans* specifies a stalk cell phenotype. *Dll4* heterozygosity induces more tip cells and sprouting angiogenesis in the leading retinal edge, and less regression in the central retina (13, 27). Increased Ki67 staining in lung EC of *Dll4^+/lacZ^* mice further supports the proliferative, nondirectional nature of the EC angiogenic behavior. As lung development progressed, the earlier dysmorphic arborization progressed to decreased capillary thickness and fewer intermediate (10–150 μM) size blood vessels, suggesting that the earlier “nonproductive” angiogenesis phenotype disrupts microvascular growth during alveolarization. These data evince that DLL4-regulated angiogenesis is required for microvascular growth and arborization from the canalicular stage of lung development.

We examined the mechanisms by which *Dll4* deficiency programs dysmorphic vascular development. *Dll4* heterozygosity induced lung EC *Vegfa* and *Nrp1* expression. Increased VEGFA expression in *Dll4^+/LacZ^* mice could be a regulatory autocrine mechanism related to decreased DLL4 as described in the retina (13). Whether the combination of increased *Vegfa* and decreased *Dll4* in *Dll4^+/lacZ^* EC dysregulates VEGFA-DLL4 gradient-specified EC tip cell migration during sprouting angiogenesis needs to be studied. Previous studies have shown that increased NRP1 in the presence of increased VEGFA induces angiogenesis (28). Our results showing exaggerated angiogenesis in *Dll4^+/lacZ^* mice in the presence of increased *Vegfa* and *Nrp1* is consistent with the above. Interestingly, we also found that *Jag1* was strongly induced in mouse lung EC of *Dll4^+/lacZ^* mice. *Dll4* and *Jag1* serve opposing functional roles in regulating angiogenesis in retina, although both of them are Notch1 ligands (29). *Dll4* represses angiogenesis, whereas *Jag1* stimulates angiogenesis in the retina. EC *Jag1* deficiency disrupts vascularization, whereas *Jag1* overexpression induces exaggerated angiogenesis in the retina (29). Our lung data showing exaggerated angiogenesis in association with increased EC JAG1 in *Dll4^+/lacZ^* mice are consistent with retinal data. Increased NICD expression in *Dll4^+/lacZ^* lung EC may be driven by JAG1, and potentially induces a different EC transcriptional program compared with DLL4-NICD signaling. In summary, our data show that lack of *Dll4* induces *Jag1*, *Nrp1,* and NICD, which in the presence of increased *Vegfa* programs aberrant hypersprouting angiogenic phenotype. In contrast to the retina, a recent study by Vila Elis et al. suggested that traditional markers of EC tip versus stalk cell specification cannot be defined in the alveolar niche (13, 20, 23). Alveolar capillary EC expressing *Car4* under AT1 VEGF stimulation (*Plvap*^–^*, Apln^+^*) exhibit tip cell–like behavior, whereas alveolar capillary EC that do not express *Car4* (*Plvap^+^, Aplnr^+^*) exhibit more stalk cell–like behavior. Our data show that Dll4 haploinsufficiency decreases EC expression of *Car4* and *Apln*, whereas *Aplnr* expression increases, suggesting a polarization toward stalk cell–like behavior. Studies examining the relationship between VEGF, CAR4, and DLL4 in regulating angiogenesis, EC specification, and AT1 interactions will be pursued in the future.

To identify novel, lung-specific mechanisms that could program deviant vascularization in *Dll4^+/lacZ^ mice* we performed RNA-Seq. Genes reported to regulate pathological angiogenic signaling increased, whereas genes that suppress angiogenesis were decreased in *Dll4^+/lacZ^ mice*. *Foxn4* and *Thbs4* are proangiogenic by regulating Notch and EC migration (30, 31), whereas *Ccl2* and *Lrg1* are NF-κB–regulated genes implicated in inflammatory angiogenesis (32, 33). *Stat1*, *Klf2*, and *Il17f* expression, which suppress angiogenesis in pathological conditions, was inhibited (34–37). Whether these pathways are specific to the lung or mediate deviant angiogenesis phenotypes described in the retina or embryogenesis with DLL4 deficiency remains unknown. Although the mechanistic relationships between DLL4 and novel targets identified to the overall vascular phenotype in *Dll4^+/lacZ^ mice* need to be studied, these data suggest that *Dll4* haploinsufficiency programs a dysregulated angiogenic phenotype in the lung.

To investigate whether the effect of *Dll4* deficiency on aberrant angiogenesis is cell autonomous and is relevant to human primary lung EC, we pursued studies in HLEC. We immortalized primary HLEC to HLEC-Im to investigate the effect of permanent Dll4 deficiency on angiogenic behavior in robust 3D-angiogenesis assays. HLEC-Im with 50% *DLL4* knockdown demonstrated exuberant endothelial sprouting, with disorganized network growth and branching, consistent with in vivo phenomenon noted in *Dll4^+/lacZ^* mouse lung EC. Studies in primary fetal HLEC using transient siRNA-based knockdown also revealed similar effects with exaggerated branching and network formation with partial Dll4 deficiency. We also noted that transient *DLL4* silencing upregulated JAG1, NICD, and HES1 in primary HLEC, consistent with our in vivo data in *Dll4^+/lacZ^* mice. Interestingly, silencing JAG1 induced DLL4 expression, suggesting reciprocal inhibition between the 2 NOTCH ligands. However, silencing JAG1 or both JAG1 and DLL4 suppressed NICD, suggesting JAG1 is required for NICD induction in HLEC. These data indicate that mechanisms underlying deviant angiogenesis with *DLL4* deficiency are operational in fetal lung EC and are cell autonomous.

As DLL4 deficiency programs deviant vascularization, we posited that DLL4 is required for lung alveolarization. DLL4 was expressed in the alveolar septum of human lungs, and C57BL/6 and *Dll4^+/lacZ^* mice, both at the base and leading EC of the alveolar septum. At E17.5, we noted that whereas distal lung acinar development was progressing in *Dll4^+/+^* mice, there was an immature glandular structure in *Dll4^+/lacZ^* littermates. At the early alveolar stage (P6), we observed double capillary loops in the primary septa of *Dll4^+/+^* mice; however, this was inconsistently observed in *Dll4^+/lacZ^* mice. Secondary septal development was impaired in *Dll4^+/lacZ^* lungs, as noted by shorter, blunted (ghost) alveolar septa in *Dll4^+/lacZ^* mice. Morphometry revealed disrupted lung development from the saccular phase, and gender-independent impaired alveolarization in the mid-alveolar phase and on lung maturity in *Dll4^+/lacZ^* mice. Lung transcriptome profiling discovered that transcriptional regulators of AT1 maturation (*Esr2* and *Klf2*) progressively decreased in *Dll4^+/lacZ^* mice. Esr2 is highly expressed in both AT1 and AT2 cells, and *Esr2^–/–^* mice have impaired alveolarization (38). KLF2 is a known regulator of AT1 and lung development (39). Gene expression of AT1 markers, such as Aqp5, Pdpn, and Hopx, decreased progressively during postnatal lung development in *Dll4^+/lacZ^* mice. AQP5 IF staining showed less continuously distributed AT1 in *Dll4^+/lacZ^* mice in comparison with *Dll4^+/+^* mice, confirming defective AT1 development/maturation. Recent literature suggest that a diverse array of biological molecules secreted by EC facilitate organ development, i.e., “angiocrine signaling”. Further investigation may lend clues to how Dll4-dependent EC secretion of angiocrine factors regulate AT1 development or maturation (40). Interestingly, targeting AT2 lineage showed that RNA, protein, and SFTPC^+^ cells were increased with *Dll4* haploinsufficiency. Similarly, PDGFRA^+^ cells that mark myofibroblasts, and are critical for septal formation, were increased in *Dll4^+/lacZ^* mice (41). This was confirmed by increased protein and RNA expression in the postnatal lung. We speculate that PDGFRA^+^ signaling induction represents a counterregulatory mechanism to compensate for impaired septal development with Dll4 hemizygosity. Although demonstrating the importance of EC DLL4 signaling to several lung lineages, it is outside the scope of the current study to investigate this complex biology.

This is the first study to our knowledge to demonstrate that DLL4 regulates microvascular growth and arborization during distal lung ontogeny, and *Dll4* deficiency programs deviant angiogenesis disrupting lung vascular patterning and alveolarization. These phenotypic changes occurred concurrently with altered expression signatures of lung EC fate specification and Notch signaling, as well as impaired AT1 lineage marker expression. Studies employing drug-dependent VEGF-signaling blockade or VEGF rescue in experimental models of developing lung injury demonstrated the importance of VEGF in lung development (4, 42, 43). These studies postulated “the vascular hypothesis,” i.e., vascular injury in the developing lung programming defective alveolarization (4). Our results support the “vascular hypothesis” from a lung development perspective by indicating that Dll4, an EC Notch ligand, is obligately required for distal lung morphogenesis. We also demonstrate that hyperoxia, a major risk factor for BPD in premature infants, inhibits DLL4 expression in the developing mouse lung and human lung EC indicating a potential role for DLL4 in BPD. Future studies will examine crosstalk between EC DLL4 and other cell types required for distal lung development, and the relevance of deviant DLL4 signaling to impaired vascular development in BPD affecting preterm infants.

## Methods

### Antibodies and primers.

Antibodies for staining: mouse anti-DLL4 (sc-365429, SCBT) and anti-PECAM (sc-376764, SCBT); rabbit anti-ERG (ab92513, Abcam), anti-AQP5 (orb15125, Biorbyt), anti-APLNR (20341-1-AP, Thermo Fisher Scientific), anti-SPC (ab90716, Abcam), anti-PDGFRA (ab203491, Abcam), anti-JAG1 (ab7771, Abcam); rat anti-PECAM (550274, BD Biosciences), anti-Ki67 (11-5698-82, Thermo Fisher Scientific); goat anti-Carbonic Anhydrase IV (CAR4) (PA5-47312, Thermo Fisher Scientific); and chicken anti-β galactosidase (ab9361, Abcam). Antibodies for Western blot: mouse anti-DLL4 (sc-365429, SCBT), anti-HES1 (sc-166410, SCBT), anti-JAG1 (sc-390177, SCBT), anti-PECAM (sc-376764, SCBT), anti-NR2F2 (sc-271940, SCBT); rabbit anti-NICD (4147, Cell Signaling), anti-PDGFRA (ab203491, Abcam), anti-SPC (ab90716, Abcam), and anti-VEGFA (ab46154, Abcam); goat anti-DLL4 (ab7280, Abcam); anti-mouse β-actin (ACTB) (A1978, Sigma). The primers were purchased commercially from Sigma.

### Mouse model.

Care of mice during experimental procedures was conducted in accordance with the policies at the University of Missouri–Kansas City Lab Animal Resource Center (Kansas City, Missouri, USA) and the NIH guidelines for the care and use of laboratory animals. Protocols had prior approval from the University of Missouri–Kansas City IACUC. *Dll4^+/lacZ^* CD1 (*Dll4^+/–^*) mouse strain was rederived from cryofrozen sperm with permission from Janet Rossant (Department of Molecular and Medical Genetics, University of Toronto, Toronto, Canada). WT C57BL/6 and CD1 mice were obtained commercially from Charles River. Our experiments did not involve surgical procedures. Mice were euthanized using 100 mg/kg i.p injection pentobarbital, exsanguinated after cessation of heartbeat, and the lungs were harvested.

### Cell culture and immortalized HLEC generation.

Primary HLECs were purchased from ScienCell (HPMEC, 3000) and grown as described in previous studies (44). The lots used from ScienCell corresponded to fetal 18-week-old male or female. We followed the company’s protocol and immortalized HLEC with Lentivirus containing SV40 large T antigens (ABM). The in vitro angiogenesis network formation assay on Matrigel using primary HLECs was performed as previously described (45). 3D angiogenesis assay was performed as previously described (46). A total of 2.5 ng/mL VEGF was added but without fibroblasts. Beads coated with immortalized HLEC were embedded onto a 3D gel and cultured in 24-well plates for 4 days. A total of 1–4 beads per well, 8 wells per group, were counted for 3D angiogenesis. HLECs were cultured in a sealed chamber containing 85% O_2_ and 5% CO_2_ for hyperoxia treatment.

### Isolation of murine lung ECs.

Mouse lung ECs were extracted from whole lung tissues as done previously (44).

### siRNA-mediated gene silencing.

siRNA sequences targeting human *DLL4* (si*DLL4*) and *JAG1* (si*JAG1*) were purchased from Santa Cruz Biotechnology (SCBT), and gene silencing was performed as per the manufacturer’s recommendations (44). For the nonsilenced cells, control siRNA (consisting of scrambled sequence that does not interfere with cellular function) was used (SCBT).

### shRNA-mediated DLL4 gene knockdown.

For immortalized HLEC (HLEC-Im), lentivirus containing *DLL4* *shRNA* was generated and used to knockdown *DLL4* expression. For the nonsilenced cells, control shRNA that does not interfere with cellular function was used (Addgene).

### Quantification of capillary diameter and intermediate blood vessels.

For determining capillary diameter, we randomly chose 3 fields and measured 6 capillaries per field with Aperio ImageScope software (Leica Biosystems) across experimental groups (*n* = 5 in each group). For determining intermediate blood vessel numbers, we randomly chose 4 fields per mouse (sections) across experimental groups (*n* = 5 in each group) and counted the number of blood vessels sized between 10 and 150 μm. The data were analyzed using 1-way ANOVA.

### IHC, IF, and X-gal staining.

IHC and IF was done as in our previous study (44). The lungs of the mouse pups were fixed in glutaraldehyde and frozen, and sections were cut onto slides for X-gal staining. The frozen slides used for X-gal staining were fixed in glutaraldehyde for 10 minutes and then incubated in X-gal staining buffer at 37°C for 1–2 hours. The slides were fixed in formalin if IHC staining was needed after X-gal staining. The slides were counterstained in Nuclear Fast Red (Sigma) and mounted in Permount Mounting Medium. Human lung tissue from different developmental stages obtained from human autopsy specimens housed within the Pathology Department of Children’s Mercy Hospital were used for IF staining. Whole-mount staining of E14.5 lung followed a published method (47). Primary antibodies are previously described. A sample size of *n* ≥ 3 was used for the human and mouse sample/group.

### Quantification of SPC^+^, Ki67^+^, and PDGFRA^+^ cells.

The sections of mouse lungs were prepared and stained as previously described. At least 5 mice were in each experimental group, and quantification of the percentage of SPC/DAPI, PDGFRA/Harris Hematoxylin, Ki67/ERG per high-power field (HPF) are reported as an average of 5 images taken per mouse.

### Quantification of mRNA expression using RT-PCR.

Total RNA was extracted, and qRT-PCR was carried out as in the previous study (44). β-Actin and *18S* were used as housekeeping genes. The relative gene expression was calculated using the Pfaffl Method (48).

### Immunoblotting for quantifying protein expression.

Immunoblotting was carried out following standard protocols (44). Densitometry was performed using ImageJ Software (NIH), and changes were normalized to ACTB or the corresponding nonphosphorylated protein.

### H&E staining to assess lung development in inflated mouse lungs.

CD1 and C57Bl6 mouse pups were euthanized on P8, P14, and P28. The lungs were inflated using a fixed volume of formalin as previously described (44). Radial alveolar counts and mean linear intercepts were quantified as before (49). For P2 lungs, uninflated lungs were used for quantification of distal air space development.

### RNA-Seq.

*Dll4^+/+^* and *Dll4^+/lac^* pups were euthanized using 100 mg/kg i.p injection pentobarbital at P6. Total cellular RNA was isolated from lungs using the MirVana kit (Thermo Fisher Scientific) according to the manufacturer’s instructions. Poly-A selected NEBNext Ultra RNA Libraries (New England BioLabs) were sequenced on an Illumina NovaSeq platform using a 150-bp paired-end sequencing strategy (Novogene Corporation). Each sample was sequenced to an average of 52.8 million reads with >94% of bases above Q30. Mapping of RNA-Seq reads and transcript assembly and abundance estimation were conducted and reported in Fragments Per Kilobase of exon per million fragments mapped (FPKM). To identify genes that were differentially expressed between heterozygous and WT mice, the FCs for each gene were calculated by dividing the average FPKM for the heterozygous mice by the average FPKM for the WT mice. Sample clustering was performed on log2-transformed FCs with the heatmap.2 R function from the R gplots package, using default parameters (Euclidian distance and “complete” clustering method). The differentially expressed genes were analyzed using Ingenuity Pathway Analysis (IPA, Ingenuity Systems Inc.). The data discussed in this publication have been deposited in NCBI’s Gene Expression Omnibus (50) (GEO accession GSE164732; https://www.ncbi.nlm.nih.gov/geo/query/acc.cgi?acc=GSE164732).

### scRNA-Seq and Dll4 expression plot.

Three C57Bl6 pups were euthanized using 100 mg/kg i.p. injection pentobarbital at P14. Fresh lung single-cell digests furnished 10^4^ cells (19), which were loaded on a 10x Genomics v3 scRNA chip using 10× Genomics v3 scRNA reagents. Library preps were done, and completed libraries were sequenced on an Illumina NovaSeq 6000 instrument with 25,000 reads/cell targeted. Raw sequencing data were processed through bcl2fastq2 (Illumina), resulting in 2 FASTQ files per library. Individual 10× libraries were processed through Cell Ranger v3.0.2, and the resulting count matrices were imported into Seurat v3.1.0 for normalization and clustering following Seurat’s SCTransform-based workflow. SingleR was used to assign a predicted cell type to each cluster. *Dll4* expression was plotted for all cells from RA-exposed mice using the SCTransform normalized counts generated by Seurat. The data discussed in this publication have been deposited in NCBI’s Gene Expression Omnibus (50) (GEO accession GSE164621; https://www.ncbi.nlm.nih.gov/geo/query/acc.cgi?acc=GSE164621).

### Statistics.

Data are shown as mean ± SD or median with IQR, and a *P* value of less than 0.05 was considered significant. For cell culture experiments, data from a minimum of 3 independent experiments with adequate technical replicates were used for quantification. All animal data were obtained in littermate controls. For animal experiments, a minimum of 5 animals were used for each experimental group. For histological quantification at least 2 slides with 3 replicates per mice were used. RNA quantification and PCR results had 2–3 technical replicates. We initially examined whether distribution of data was Gaussian using the D’Agostino-Pearson omnibus normality test. If data were normally distributed, then 1-way ANOVA with Tukey’s post hoc test with multiple comparisons was used for analysis. For analysis with one comparison and normal distribution, the 2-tailed *t* test with Welch’s correction was used. If data did not meet Gaussian assumptions, a 2-tailed Mann-Whitney *U* test was used for analysis. For most analysis, fold changes were calculated related to expression/changes in untreated controls. Statistical analysis was done using Graphpad Prism version 7.0.

### Study approval.

Lab experiments were reviewed and approved under the University of Missouri–Kansas City Institutional Biosafety Committee, protocol number 18-28. Animal experiments were reviewed and approved under the University of Missouri–Kansas City IACUC, protocol number 1510-02.

## Author contributions

SX designed the project, conducted experiments, processed data, interpreted results, and wrote the manuscript. HLM prepared mice, performed immunoblotting and qRT-PCR, processed data, and prepared the figures. NT performed morphological analysis and DLL4 mapping. SM bred and genotyped mice, performed H&E staining, and performed mouse lung morphological analysis. JJ analyzed scRNA-Seq data. DH analyzed *Dll4* RNA-Seq data. MN and TK provided insight into certain aspects of study, helped with manuscript revision, and edited the manuscript. VS was responsible for overall conception of the project, including developing experimental design, interpreting results, and writing and editing the manuscript.

## Supplementary Material

Supplemental data

## Version 1 04/08/2021

Electronic publication
