# Supplementary material for: Delta-like 4 is required for pulmonary vascular arborization and alveolarization in the developing lung
Source: JCI Insight. 2021 Apr 8;6(7):e134170. doi: 10.1172/jci.insight.134170 (PMC8119184; doi:10.1172/jci.insight.134170)

Supplemental Table 1

Supplemental Table 1. Major lung transcriptome networks dysregulated in *Dll4* $^{+/lacZ}$ pups in comparison with litter-mate *Dll4* $^{+/+}$ mice on postnatal day 6, identified by RNA sequencing.

Categories	Diseases/Functions Annotation	p-Value	Activation z-score
**Non-cardiovascular pathways**			
Endocrine System Disorders, Gastrointestinal Disease, Immunological Disease, Metabolic Disease, Organismal Injury and Abnormalities	Insulin-dependent diabetes mellitus	8.85E-10	
Cancer, Endocrine System Disorders, Organismal Injury and Abnormalities	Endocrine carcinoma	6.53E-09	
Cancer, Gastrointestinal Disease, Organismal Injury and Abnormalities	Gastrointestinal adenocarcinoma	5.65E-08	
Antimicrobial Response, Inflammatory Response	Antimicrobial response	0.000000363	-1.154
Immunological Disease	Systemic autoimmune syndrome	0.00000039	
Metabolic Disease	Glucose metabolism disorder	0.00000067	-1.064
Cell-To-Cell Signaling and Interaction, Cellular Assembly and Organization	Cell-cell contact	0.00000069	1.284
**Cardiovascular Pathways**			
Cardiovascular System Development and Function, Tissue Morphology	Morphology of vein	0.000776	
Cardiovascular System Development and Function, Cell-To-Cell Signaling and Interaction	Activation of endothelial cells	0.00276	
Cardiovascular System Development and Function	Development of vasculature	0.00574	2.372
Cardiovascular System Development and Function, Ophthalmic Disease, Organismal Development, Injury and Abnormalities, Visual System Development and Function	Neovascularization of choroid	0.00715	
Cardiovascular System Development and Function, Organismal Development	Angiogenesis	0.00825	2.372

Cardiovascular System Development and Function, Connective Tissue Development and Function, Organismal Development, Tissue Morphology	Quantity of pericytes	0.00878	
Cardiovascular System Development and Function, Organismal Development, Tissue Morphology	Quantity of microvessel	0.0104	

Supplemental Figure 1

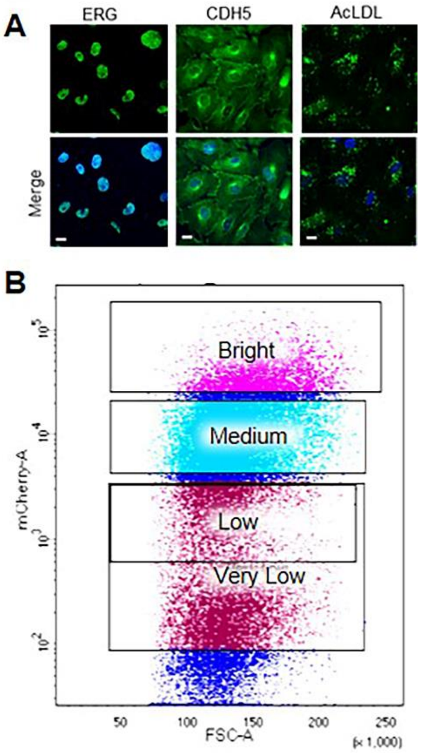

Supplemental Figure 2

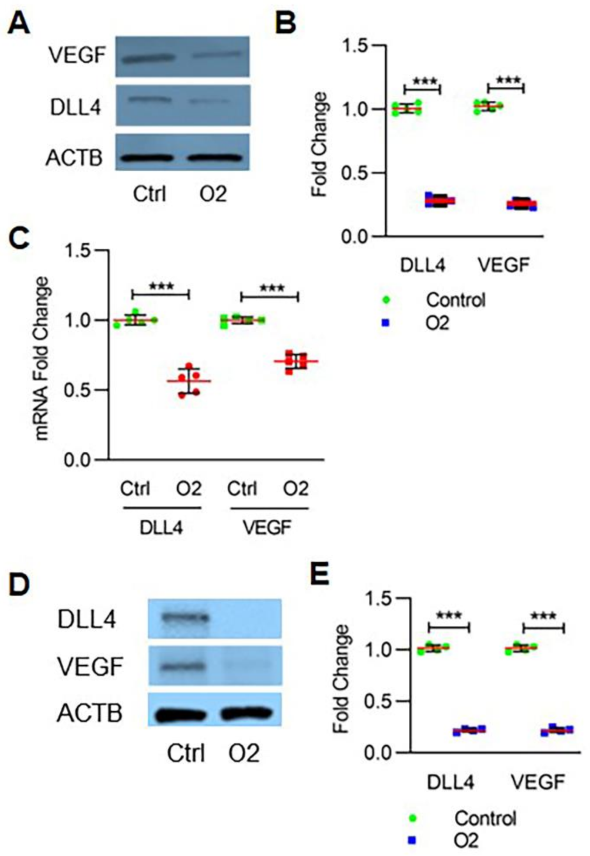

Supplement: Supplemental data [file jciinsight-6-134170-s195.pdf]
